# Adiabatic Amplification of Energy and Magnetic Moment of a Charged Particle after the Magnetic Field Inversion

**DOI:** 10.3390/e25040596

**Published:** 2023-03-31

**Authors:** Viktor V. Dodonov, Alexandre V. Dodonov

**Affiliations:** 1 Institute of Physics, University of Brasilia, P.O. Box 04455, Brasilia 70919-970, DF, Brazil; 2 International Center of Physics, University of Brasilia, Brasilia 70919-970, DF, Brazil

**Keywords:** generalized adiabatic invariants, generalized Born–Fock theorem, time-dependent Larmor frequency, mean energy, energy fluctuations, mean value and fluctuations of magnetic moment, single and multiple frequency passages through zero

## Abstract

We study the evolution of the energy and magnetic moment of a quantum charged particle placed in a homogeneous magnetic field, when this field changes its sign adiabatically. We show that after a single magnetic field passage through zero value, the famous adiabatic invariant ratio of energy to frequency is reestablished again, but with a proportionality coefficient higher than in the initial state. The concrete value of this proportionality coefficient depends on the power index of the frequency dependence on time near zero point. In particular, the adiabatic ratio of the initial ground state (with zero radial and angular quantum numbers) triplicates if the frequency tends to zero linearly as a function of time. If the Larmor frequency attains zero more than once, the adiabatic proportionality coefficient strongly depends on the lengths of the time intervals between zero points, so that the mean energy behavior can be quasi-stochastic after many passages through zero value. The original Born–Fock adiabatic theorem does not work after the frequency passes through zero. However, its generalization is found: the initial Fock state becomes a wide superposition of many instantaneous Fock states, whose weights do not depend on time in the new adiabatic regime.

## 1. Introduction

We consider the motion of a non-relativistic spinless particle of mass M=1 and charge *e* in the xy-plane in the presence of a uniform time-dependent magnetic field B(t) directed along the *z*-axis (perpendicular to the plane). Since this motion is independent from the motion along the axis, it can be described by means of the two-dimensional Hamiltonian (in the Gauss system of units)

(1) $$H\left(t\right)=\frac{1}{2}\sum_{j=1,2}{\left[{\hat{p}}_{j}-eA_{j}\left(t\right)/c\right]}^{2}.$$

Here, $p_{j}$ and $A_{j}$ are components of the canonical momentum and vector potential, respectively. In this paper, we assume the “circular” gauge of the vector potential, A(t)=B(t)(−y,x)/2. Then,

(2) $$H\left(t\right)=\frac{1}{2}\left[{\hat{p}}_{x}^{2}+{\hat{p}}_{y}^{2}+\omega^{2}\left(t\right)\left({\hat{x}}^{2}+{\hat{y}}^{2}\right)\right]-\omega \left(t\right)\left(\hat{x}{\hat{p}}_{y}-\hat{y}{\hat{p}}_{x}\right),\;\omega \left(t\right)=eB\left(t\right)/\left(2c\right).$$

Solutions to the stationary Schrödinger equation $\hat{H}\psi =E\psi$ with Hamiltonian (2) (including an additional isotropic harmonic oscillator potential) were found for the first time by Fock [1] and later by Darwin [2] in the special case of B(t)=const. These solutions can be written in polar coordinates as follows,

(3) $$\psi_{n_{r}m}\left(r,\varphi \right)=\sqrt{\frac{\kappa n_{r}!}{\pi \left(n_{r}+\left|m\right|\right)!}}{\left(\kappa r^{2}\right)}^{|m|/2}L_{n_{r}}^{\left(|m|\right)}\left(\kappa r^{2}\right)\exp\left(-\kappa r^{2}/2+im\varphi \right),$$

(4) $$\kappa =\left|\omega \right|/\hbar ,\;m=0,\pm 1,\pm 2,\ldots ,\;n_{r}=0,1,2,\ldots .$$

Function $L_{n}^{\left(\alpha \right)}\left(z\right)$ is the generalized Laguerre polynomial, defined as [3,4]

$$L_{n}^{\left(\alpha \right)}\left(z\right)=\frac{1}{n!}e^{z}z^{-\alpha }\frac{d^{n}}{dz^{n}}\left(e^{-z}z^{\alpha +n}\right).$$

The energy eigenvalues are given by the formula

(5) $$E_{n_{r}m}=\hbar \left|\omega \right|\left(1+\left|m\right|+2n_{r}\right)-\hbar \omega m,$$

which consists of two parts, in accordance with the two parts of Hamiltonian (2). Note that the radial part of the wave function (3) depends on the absolute value |m| of the canonical angular momentum eigenvalue. The algebraic value *m* (which can be either positive or negative) enters through the preserved canonical angular momentum operator in Hamiltonian (2). If ω>0, all states with m≥0 have the same energy $\hbar |\omega |\left(1+2n_{r}\right)$, meaning an infinite degeneracy of these energy levels. On the other hand, the same infinite degeneracy happens for all states with m≤0 if ω<0. These observations are important for the interpretation of results in the following sections.

What happens with the solution (3), when the magnetic field depends on time after some initial time instant $t_{i}$? A general answer was given many years ago by Malkin, Man’ko and Trifonov [5]:

(6) $$\Psi_{n_{r}m}\left(r,\varphi ;t\right)=\sqrt{\frac{K\left(t\right)n_{r}!{\left[K\left(t\right)r^{2}\right]}^{\left|m\right|}}{\pi \left(n_{r}+\left|m\right|\right)!}}L_{n_{r}}^{\left(|m|\right)}\left[K\left(t\right)r^{2}\right]\exp\left(i\frac{\dot{\varepsilon }}{2\hbar \varepsilon }r^{2}+im\varphi +i\chi \left(t\right)\right).$$

The real function $K\left(t\right)=\hbar^{-1}{\left|\varepsilon \left(t\right)\right|}^{-2}$ is determined by the complex function ε(t), which is the solution to the classical equation of the harmonic oscillator with a time-dependent frequency,

(7) $$\ddot{\varepsilon }+\omega^{2}\left(t\right)\varepsilon =0,$$

satisfying the initial conditions

(8) $$\varepsilon \left(t_{i}\right)={\left[\omega \left(t_{i}\right)\right]}^{-1/2},\;\dot{\varepsilon }\left(t_{i}\right)=i{\left[\omega \left(t_{i}\right)\right]}^{1/2}.$$

These conditions fix the value of the time-independent Wronskian

(9) $$\dot{\varepsilon }\varepsilon^{*}-{\dot{\varepsilon }}^{*}\varepsilon =2i.$$

We assume that $\omega (t_{i})>0$. Since the phase χ(t) is not important for our purposes, we do not provide herein its explicit (rather complicated) expression. Functions (6) are orthogonal and normalized:

(10) $$\langle \Psi_{n_{r}^{\prime }m^{\prime }}\left(t\right)|\Psi_{n_{r}m}\left(t\right)\rangle =\delta_{mm^{\prime }}\delta_{n_{r}^{\prime }n_{r}}.$$

Various results related to quantum systems described by means of time-dependent Hamiltonian (2) were found over the past five decades after paper [5]: see, e.g., papers [6,7,8,9,10,11,12,13,14]. However, an interesting special case of time inversion of the magnetic field, especially in the adiabatic regime, was not considered in previous studies, despite adiabatic approximation in quantum mechanics being the subject of numerous papers: see, e.g., [15,16,17,18,19,20,21,22]. The aim of our paper is to fill in this gap (at least partially). Remember that stationary homogeneous magnetic fields can be created inside solenoids with constant currents. On the other hand, alternating currents are quite common in our daily lives. Hence, it can be interesting to understand, what can happen with initial quantum states of a charged particle placed inside a solenoid with an alternating current. It is worth noticing in this connection, that periodic variations of the magnetic field created by a current with the standard frequency 50 Hz can be considered as adiabatic, if the amplitude of the magnetic field is sufficiently large. Indeed, the Larmor frequency of an electron in the magnetic field of the order of 1 T is of the order of $10^{11}$ s${}^{-1}$. This means that the period of rotation of the particle is many orders of magnitude smaller than the scale of variations of the magnetic field. This presents one of the motivations behind our study.

The adiabatic evolution of the unidimensional harmonic oscillator, whose frequency passes through zero, was studied in the recent paper by Ref. [23]. However, when the frequency turns into zero and returns to nonzero values for this system, the situation seems to be rather exceptional (although possible). On the other hand, a passage of the Larmor frequency through zero is quite natural if the magnetic field changes its sign. In addition, the existence of the second degree of freedom and additional invariant (the canonical angular momentum) adds more interesting features to the dynamics of the system described by means of Hamiltonian (2). One of these new features is the evolution of the magnetic moment. Another interesting feature is the infinite degeneracy of the stationary energy levels in the presence of a magnetic field. An analysis of these additional features represents the second motivation of the present study.

The remainder of this paper is structured as follows. In Section 2, we bring general formulas describing the adiabatic evolution of the Fock states and the magnetic moment, including the adiabatic regimes with and without crossing zero value of the Larmor frequency. Section 3 and Section 4 are devoted to an analysis of the new adiabatic parameters in the cases of single and multiple transitions of the frequency through zero. In Section 5, we consider the violation and generalization of the Born–Fock theorem when a magnetic field slowly changes its sign. Analytical results are illustrated with several figures. In Section 6, we study the evolution of the mean energy and the mean magnetic moment (as well as their fluctuations) in the case of initial Fock states. A more general case of initial “invariant states” of the magnetic moment operator is considered in Section 7. Section 8 provides the discussion of main results.

## 2. Adiabatic Evolution of the Fock States

The adiabatic (quasiclassical) approximate complex solution to Equation (7) with ω(t)>0 can be written in the form

(11) $$\varepsilon \left(t\right)\approx {\left[\omega \left(t\right)\right]}^{-1/2}e^{i\tilde{\phi }\left(t\right)},\;\dot{\varepsilon }\left(t\right)\approx i{\left[\omega \left(t\right)\right]}^{1/2}e^{i\tilde{\phi }\left(t\right)},\;\tilde{\phi }\left(t\right)=\int_{t_{i}}^{t}\omega \left(z\right)dz.$$

In this approximation, the solution (6) assumes the form (3) with the instantaneous value of function ω(t). This result agrees with the famous Born–Fock adiabatic theorem [15]. The mean energy evolves according to Equation (5) with ω=ω(t). In particular, all states with m≥0 have the same mean energy $\hbar \omega \left(t\right)\left(1+2n_{r}\right)$ if ω(t)>0.

The magnetic moment operator has the form [24,25,26,27,28,29,30,31,32]

(12) $$\hat{M}=\frac{e}{2c}\left(\hat{x}{\hat{\pi }}_{y}-\hat{y}{\hat{\pi }}_{x}\right),\;{\hat{\pi }}_{j}\equiv {\hat{p}}_{j}-eA_{j}\left(\hat{x},\hat{y}\right).$$

For the “circular” gauge of the vector potential, A=B(−y,x)/2, we can write

(13) $$\hat{M}=\left[\hat{x}{\hat{p}}_{y}-\hat{y}{\hat{p}}_{x}-\omega \left({\hat{x}}^{2}+{\hat{y}}^{2}\right)\right]e/\left(2c\right).$$

The magnetic moment operator does not commute with Hamiltonian (2):

(14) $$\left[\hat{H},\hat{M}\right]=i\omega \mu_{B}\left(\hat{x}{\hat{\pi }}_{x}+{\hat{\pi }}_{x}\hat{x}+\hat{y}{\hat{\pi }}_{y}+{\hat{\pi }}_{y}\hat{y}\right),$$

where $\mu_{B}=e\hbar /\left(2c\right)$ is the Bohr magneton (for the unit mass chosen in this paper).

The mean value $\langle r^{2}\rangle \equiv \langle x^{2}+y^{2}\rangle$ in the state (6) is given by a simple formula (see Appendix A)

(15) $${\langle r^{2}\rangle }_{n_{r},m}={\left[K\left(t\right)\right]}^{-1}\left(2n_{r}+\left|m\right|+1\right).$$

Consequently, the mean value of the magnetic moment in the Fock state (6) equals

(16) $${\langle M\rangle }_{n_{r},m}=\mu_{B}\left[m-{\omega \left(t\right)|\varepsilon \left(t\right)|}^{2}\left(2n_{r}+\left|m\right|+1\right)\right].$$

This is not an eigenvalue of the magnetic moment operator, since the spectrum of this operator is continuous [33]. Formula (16) is interesting because it shows that the *mean value* of the magnetic moment in the energy eigenstate (when ω=const>0 and ${\omega |\varepsilon |}^{2}=1$) is proportional to the energy eigenvalue, although non-commuting operators $\hat{H}$ (2) and $\hat{M}$ (13) have quite different structures and spectra.

Fluctuations of the magnetic moment, characterized by the variance $\sigma_{M}=\langle {\hat{M}}^{2}\rangle -{\langle \hat{M}\rangle }^{2}$, can be calculated with the aid of formula (see Appendix A)

(17) $${\langle r^{4}\rangle }_{n_{r},m}={\left[K\left(t\right)\right]}^{-2}\left[6n_{r}\left(n_{r}+\left|m\right|+1\right)+\left(|m|+1\right)\left(|m|+2\right)\right].$$

The result is

(18) $$\sigma_{M}^{\left(n_{r},m\right)}={\left[\mu_{B}\omega \left(t\right){\left|\varepsilon \left(t\right)\right|}^{2}\right]}^{2}\left[2n_{r}\left(n_{r}+\left|m\right|+1\right)+\left|m\right|+1\right].$$

We see that fluctuations of the magnetic moment can be strong in the initial energy eigenstates (when ${\omega |\varepsilon |}^{2}=1$) with large values of the quantum numbers $n_{r}$ and |m|. However, they are strong in the ground state ($n_{r}=m=0$) as well: $\sigma_{M}^{\left(0,0\right)}={\left[{\langle M\rangle }_{0,0}\right]}^{2}$.

In the adiabatic regime (11), the mean magnetic moment does not depend on time, providing an example of adiabatic invariants:

(19) $${\langle M\rangle }_{n_{r},m}=\mu_{B}\left[m-\left(2n_{r}+\left|m\right|+1\right)\right]=-E_{n_{r},m}\left(t\right)/\left[\hbar \omega \left(t\right)\right].$$

However, the solution (11) holds under the condition

(20) $$|\dot{\omega }|/\omega^{2}\left(t\right)\ll 1,$$

which is clearly broken when ω(t)=0. Nonetheless, when the frequency slowly passes through zero value and slowly becomes not too small again, the conditions of the quasiclassical approximation are reestablished. We suppose that ω=0 at some instant $t_{*}>t_{i}$. Then, the solution to Equation (7) can be written in the following most general quasiclassical form at $t\gg t_{*}$ [this formal relation means that time instant *t* is so far from the instant $t_{*}$ that the condition (20) can be considered fulfilled]:

(21) $$\varepsilon \left(t\right)\approx {\left|\omega \left(t\right)\right|}^{-1/2}\left[u_{+}e^{i\phi (t)}+u_{-}e^{-i\phi (t)}\right],\;\phi \left(t\right)=\int_{t_{*}}^{t}\left|\omega \left(\tau \right)\right|d\tau ,\;$$

(22) $$\;\dot{\varepsilon }\left(t\right)\approx i{\left|\omega \left(t\right)\right|}^{1/2}\left[u_{+}e^{i\phi (t)}-u_{-}e^{-i\phi (t)}\right].$$

Constant complex coefficients $u_{\pm }$ must obey the condition

(23) $$|u_{+}{|}^{2}-{\left|u_{-}\right|}^{2}=1,$$

which is the consequence of Equation (9). Note the choice of the lower limit of integration in the definition of the phase function ϕ(t). This choice is insignificant for the solution in the form (11), which is represented by a single exponential function. However, when one deals with a superposition of two exponential functions in (21), the choice of the integration limits influences the phases of complex coefficients $u_{\pm }$. If the frequency passes through zero, the time instant $t_{*}$ is distinguished. Therefore, the choice of $t_{*}$ as the starting point of integration seems the most natural. To fix the values of coefficients $u_{\pm }$, we choose the function ε(t) at $t\ll t_{*}$ in the form (11), but with the phase $\tilde{\phi }\left(t\right)$ replaced with the function ϕ(t) defined as in Equation (21). Then, although ϕ(t)<0 for $t<t_{*}$, the time derivative dϕ/dt=|ω(t)| is positive.

In general, the mean magnetic moment oscillates at $t\gg t_{*}$:

(24) $${\langle M\rangle }_{n_{r},m}=\mu_{B}\left[m-\frac{\omega (t)}{\left|\omega (t)\right|}w\left(t\right)\left(2n_{r}+\left|m\right|+1\right)\right],\;$$

(25) $$w\left(t\right)=|u_{+}{|}^{2}+{\left|u_{-}\right|}^{2}+2Re\left[u_{+}u_{-}^{*}e^{2i\phi (t)}\right].$$

If ω(t)<0, the function w(t) enters Equation (25) with the positive sign. Otherwise, its contribution is negative. Due to Equation (23), we have the inequalities

(26) $$w_{min}={\left(\sqrt{1+|u_{-}{|}^{2}}+\left|u_{-}\right|\right)}^{-2}\leq w\left(t\right)\leq {\left(\sqrt{1+|u_{-}{|}^{2}}+\left|u_{-}\right|\right)}^{2}=w_{max}.$$

Since $w_{min}<1$ and $w_{max}>1$ if $|u_{-}|>0$, the mean magnetic moment (24) oscillates between negative and positive values for sufficiently large (by absolute value) negative values of the quantum number *m*.

## 3. Examples of Adiabatic Coefficients

Exact values of the adiabatic coefficients are determined by the behavior of function ω(t) near zero value. It was proposed in paper [23], that absolute values of these coefficients can be expressed in terms of the exponent *n* in the behavior $\omega^{2}\left(t\right)∼{\left|t-t_{*}\right|}^{n}$ when ω(t) passes through zero. This hypothesis was based on the analysis of exact solutions to Equation (7) for the time-dependent frequency $\omega^{2}\left(t\right)=\omega_{0}^{2}{\left|t/\tau \right|}^{n}$, when this equation can be reduced to the Bessel equation. In that paper, devoted to the adiabatic dynamics of a quantum harmonic oscillator, the exponent *n* could assume arbitrary non-negative values, as soon as function $\omega^{2}\left(t\right)$ had physical meaning. However, it is the function ω(t) that has physical meaning in the problems involving magnetic fields. For this reason, supposing that function ω(t) can smoothly change its sign, we consider here the special case of solutions obtained in [23], when *n* is an *even* number, i.e.,

(27) $$\omega \left(t\right)=\omega_{0}{\left(-t/\tau \right)}^{k},\;-\tau \leq t\leq \tau ,\;k=0,1,2,\ldots$$

Then, in the adiabatic limit $\omega_{0}\tau \gg 1$, the following formulas were derived in [23]:

(28) $$u_{+}^{-1}=\sin\left[\frac{\pi }{2(k+1)}\right],\;u_{-}=i\cot\left[\frac{\pi }{2(k+1)}\right].$$

It is probable that the most natural situation takes place for k=1, i.e., the linear time dependence of a slow transition of magnetic field through zero value. In this case, we have $|u_{-}|=1$ and $|u_{+}{|}^{2}=2$. Another interesting situation corresponds to k=2, when $|u_{-}{|}^{2}=3$. In this case, the magnetic field slowly diminishes to zero, but instead of crossing through zero, it slowly returns to the initial value. For k=3 (a “cubical” crossing through zero frequency), we have $|u_{-}{|}^{2}={\left(\sqrt{2}+1\right)}^{2}$.

Another explicit exact solution to Equation (7) was found in paper [34] for the time-dependent Larmor frequency, which changes continuously from the initial value $\omega_{i}$ to the final value $\omega_{f}$ as

(29) $$\omega \left(t\right)=\frac{\omega_{f}\exp\left(\kappa t\right)+\omega_{i}}{\exp(\kappa t)+1},\;-\infty <t<\infty ,\;\kappa >0.$$

Here, the positive parameter κ characterizes the speed of frequency evolution. The following formula was obtained in the adiabatic regime, when $|\omega_{i}-\omega_{f}|\gg \kappa /2$:

(30) $$|u_{-}{|}^{2}\approx \exp[2\pi (|{\tilde{\omega }}_{i}-{\tilde{\omega }}_{f}|-{\tilde{\omega }}_{i}-|{\tilde{\omega }}_{f}|)],\;{\tilde{\omega }}_{i,k}\equiv \omega_{i,k}/\kappa .$$

If $\omega_{i}>\omega_{f}>0$, Equation (30) yields $|u_{-}{|}^{2}\approx \exp\left(-4\pi {\tilde{\omega }}_{f}\right)\ll 1$, so that the adiabatic invariance of the magnetic moment is preserved. On the other hand, the same Equation (30) yields $|u_{-}{|}^{2}\approx 1$ for $\omega_{f}<0$ (provided $|{\tilde{\omega }}_{f}|\gg 1$). We see that two different functions ω(t), given by Equation (27) with k=1 and Equation (29), yield identical *absolute values* of the adiabatic coefficients $|u_{\pm }|$. A common feature of these two functions is the *linear* time dependence of the transition through zero value.

However, the *phases* of adiabatic coefficients are sensitive to the exact form of function ω(t). For example, it was shown in [23] that function (29) with $\omega_{f}=-\omega_{i}$ yields the complex adiabatic coefficients

(31) $$u_{+}=\sqrt{2}\;\exp\left[-4i{\tilde{\omega }}_{0}\ln\left(2\right)\right],\;u_{-}=i.$$

We see that phases of coefficients $u_{+}$ given by Equation (28) with k=1 and Equation (31) are different.

## 4. Multiple Adiabatic Passages of Magnetic Field through Zero Value

What can happen if the Larmor frequency ω(t) passes adiabatically through zero value several times, at instants $t_{0},t_{1},t_{2},\ldots$? We suppose that we know all coefficients in the transition rules through zero frequency at each instant $t_{k}$:

(32) $${\left\{{\left[\omega \left(t\right)\right]}^{-1/2}e^{i\phi_{k}\left(t\right)}\right\}}_{t\ll t_{k}}\to {\left\{{\left[\omega \left(t\right)\right]}^{-1/2}\left[u_{+}^{\left(k\right)}e^{i\phi_{k}\left(t\right)}+u_{-}^{\left(k\right)}e^{-i\phi_{k}\left(t\right)}\right]\right\}}_{t\gg t_{k}},\;$$

(33) $$\phi_{k}\left(t\right)=\int_{t_{k}}^{t}\left|\omega \left(z\right)\right|dz.$$

The resulting transformation after *N* zero-crossings can be written as

(34) $${\left\{{\left[\omega \left(t\right)\right]}^{-1/2}e^{i\phi_{0}\left(t\right)}\right\}}_{t\ll t_{0}}\to {\left\{{\left[\omega \left(t\right)\right]}^{-1/2}\left[U_{+}^{\left(N-1\right)}e^{i\phi_{0}\left(t\right)}+U_{-}^{\left(N-1\right)}e^{-i\phi_{0}\left(t\right)}\right]\right\}}_{t\gg t_{N-1}},$$

assuming that $U_{\pm }^{\left(0\right)}=u_{\pm }^{\left(0\right)}$. To establish the recurrence relations between the coefficients $U_{\pm }^{\left(N-1\right)}$ and $U_{\pm }^{\left(N\right)}$, we apply the rule (32) to the function arising after the *N*th zero, using the superposition principle and taking into account that the function ${\left[\omega \left(t\right)\right]}^{-1/2}e^{-i\phi_{k}\left(t\right)}$ transforms as $\varepsilon^{*}\left(t\right)$ after the frequency passes through zero value:

$${\left\{{\left[\omega \left(t\right)\right]}^{-1/2}e^{-i\phi_{k}\left(t\right)}\right\}}_{t\ll t_{k}}\to {\left\{{\left[\omega \left(t\right)\right]}^{-1/2}\left[u_{+}^{(k)*}e^{-i\phi_{k}\left(t\right)}+u_{-}^{(k)*}e^{i\phi_{k}\left(t\right)}\right]\right\}}_{t\gg t_{k}}.$$

Then, using the relations

(35) $$\phi_{0}\left(t\right)=\Phi_{k}+\phi_{k}\left(t\right),\;\Phi_{k}=\int_{t_{0}}^{t_{k}}\left|\omega \left(z\right)\right|dz,$$

we can write the following equation for $t\gg t_{N}$:

$$\begin{array}{ccc} U_{+}^{\left(N\right)}e^{i\phi_{0}\left(t\right)}+U_{-}^{\left(N\right)}e^{-i\phi_{0}\left(t\right)} & = & U_{+}^{\left(N-1\right)}e^{i\Phi_{N}}\left[u_{+}^{\left(N\right)}e^{i\phi_{N}\left(t\right)}+u_{-}^{\left(N\right)}e^{-i\phi_{N}\left(t\right)}\right]\\ & + & U_{-}^{\left(N-1\right)}e^{-i\Phi_{N}}\left[u_{+}^{(N)*}e^{-i\phi_{N}\left(t\right)}+u_{-}^{(N)*}e^{i\phi_{N}\left(t\right)}\right]. \end{array}$$

Equating the terms with the same time dependence $e^{\pm i\phi_{N}\left(t\right)}$, we arrive at the following recurrence relations:

(36) $$U_{+}^{\left(N\right)}=U_{+}^{\left(N-1\right)}u_{+}^{\left(N\right)}+U_{-}^{\left(N-1\right)}u_{-}^{(N)*}e^{-2i\Phi_{N}},\;$$

(37) $$U_{-}^{\left(N\right)}=U_{-}^{\left(N-1\right)}u_{+}^{(N)*}+U_{+}^{\left(N-1\right)}u_{-}^{\left(N\right)}e^{2i\Phi_{N}}.$$

One can verify that the identity $|U_{+}^{\left(N\right)}{|}^{2}-{\left|U_{-}^{\left(N\right)}\right|}^{2}=1$ is the consequence of identities

$$|U_{+}^{\left(N-1\right)}{|}^{2}-|U_{-}^{\left(N-1\right)}{|}^{2}=1,\;|u_{+}^{\left(N\right)}{|}^{2}-{\left|u_{-}^{\left(N\right)}\right|}^{2}=1.$$

We see a strong dependence of coefficients $U_{\pm }^{\left(N\right)}$ on the phases $\Phi_{N}$, i.e., on the time intervals between the passages through zero frequency. In the case of double passage through zero, the following inequalities hold:

(38) $${\left(|u_{+}^{\left(0\right)}u_{-}^{\left(1\right)}\left|-\right|u_{+}^{\left(1\right)}u_{-}^{\left(0\right)}|\right)}^{2}\leq {\left|U_{-}^{\left(1\right)}\right|}^{2}\leq {\left(|u_{+}^{\left(0\right)}u_{-}^{\left(1\right)}\left|+\right|u_{+}^{\left(1\right)}u_{-}^{\left(0\right)}|\right)}^{2}.$$

In particular, two identical passages through zero frequency can result in the value $U_{-}^{\left(1\right)}=0$, i.e., in reestablishing the standard adiabatic behavior, under the condition $Re\left[u_{+}^{\left(0\right)}e^{i\Phi_{1}}\right]=0$.

## 5. Generalization of the Born–Fock Theorem

The time-dependent solution (6) is a superposition of adiabatic eigenstates (3) with the instantaneous value of frequency ω(t) and the same value of the canonical angular momentum quantum number *m*:

(39) $$\Psi_{n_{r}m}\left(r,\varphi ;t\right)=\sum_{q_{r}=0}^{\infty }C_{n_{r}q_{r}}^{\left(m\right)}\left(t\right)\psi_{q_{r}m}\left(r,\varphi \right),\;C_{n_{r}q_{r}}^{\left(m\right)}\left(t\right)=\langle \psi_{q_{r}m}|\Psi_{n_{r}m}\left(t\right)\rangle .$$

Coefficients $C_{n_{r}q_{r}}^{\left(m\right)}\left(t\right)$ are oscillating functions of the phase ϕ(t) [defined in Equation (21)]. It is remarkable, however, that the modules squared $|C_{n_{r}q_{r}}^{\left(m\right)}{|}^{2}$ do not oscillate: they depend on the absolute values of constant parameters $u_{\pm }$ only, as shown in Appendix B:

(40) $$|C_{n_{r}q_{r}}^{\left(m\right)}{|}^{2}=\frac{\left(n_{>}+\left|m\right|\right)!n_{<}!{\left|u_{-}\right|}^{2|q_{r}-n_{r}|}}{\left(n_{<}+\left|m\right|\right)!n_{>}!{\left|u_{+}\right|}^{2(|q_{r}-n_{r}|+|m|+1)}}{\left[P_{n_{<}}^{\left(|q_{r}-n_{r}|,|m|\right)}\left(\frac{1-|u_{-}{|}^{2}}{1+|u_{-}{|}^{2}}\right)\right]}^{2}.$$

Here, $P_{k}^{\left(a,b\right)}\left(z\right)$ is the Jacobi polynomial. Other notations are as follows:

$$n_{<}=\min\left(q_{r},n_{r}\right),\;n_{>}=\max\left(q_{r},n_{r}\right).$$

Formula (40) has many interesting consequences. First, it does not depend on the sign of quantum number *m*, while the energy levels (5) are different for positive and negative values of *m*. Second, the probabilities (40) are different for different positive values of *m*, although the initial energy eigenvalues are the same, if $\omega (t<t_{*})>0$. If ω(t)<0 for $t>t_{*}$, eigenstates $\psi_{q_{r}m}\left(r,\varphi \right)$ in the expansion (39) describe the states with the instantaneous energy eigenvalues $E_{q_{r}m}\left(\omega <0\right)=\hbar \left|\omega \left(t\right)\right|\left(1+\left|m\right|+m+2q_{r}\right)$, which are different for different positive values of *m*, in contradistinction to the infinite energy levels degeneracy at $t<t_{*}$.

The simplest special case is $n_{r}=0$, when $n_{<}=0$ and $n_{>}=q_{r}$. Then, the Jacobi polynomial turns into unity, so that

(41) $$|C_{0q_{r}}^{\left(m\right)}{|}^{2}=\frac{(q_{r}+\left|m|)!\right|u_{-}{|}^{2q_{r}}}{|m|!q_{r}!{\left|u_{+}\right|}^{2(q_{r}+|m|+1)}}.$$

One can verify that $\sum_{q_{r}=0}^{\infty }{\left|C_{0q_{r}}^{\left(m\right)}\right|}^{2}=1$, due to the formula

(42) $$S_{m}\left(x\right)=\sum_{k=0}^{\infty }\frac{(m+k)!}{m!k!}x^{k}={\left(1-x\right)}^{-m-1}.$$

The mean energy in the distribution (41) can be easily calculated with the aid of Formula (42):

(43) $$\begin{array}{ccc} \langle \hat{H}\left(t\right)\rangle & = & \hbar |\omega \left(t\right)|\sum_{q_{r}=0}^{\infty }{\left|C_{0q_{r}}^{\left(m\right)}\right|}^{2}\left(1+\left|m\right|-\frac{\omega (t)}{\left|\omega (t)\right|}m+2q_{r}\right)\\ & = & \hbar |\omega \left(t\right)|\left[1+\left|m\right|-\frac{\omega (t)}{\left|\omega (t)\right|}m+2(1+|m|)|u_{-}{|}^{2}\right]. \end{array}$$

Remember that the initial energy was $E_{in}=\hbar \left|\omega_{in}\right|\left(1+|m|-m\right)$, and all initial states with m≥0 had the same energy. However, when the Larmor frequency passes through zero and changes its sign, this infinite degeneracy is removed. Moreover, the degeneracy is removed even when the frequency maintains its sign, if $u_{-}\neq 0$. In any case, the adiabatic ratio $\langle \hat{H}\left(t\right)\rangle /|\omega \left(t\right)|$ *always* increases when the frequency passes through zero.

The distribution (41) as a function of $q_{r}$ has a rather simple form. It decays monotonously if m=0, going to a distribution with a single maximum at $q_{r}+1\approx \left|m|\;\right|u_{-}{|}^{2}$ for large values of |m|. However, the situation is more intricate for nonzero values of the initial radial quantum number $n_{r}=n$. In the special case when $|u_{-}{|}^{2}=1$ and $|u_{+}{|}^{2}=2$ (a single linear transition of the magnetic field through zero), the general distribution (40) assumes the form

(44) $$\begin{array}{ccc} |C_{nq}^{\left(m\right)}{|}_{|u_{-}|=1}^{2} & = & \frac{\left(n_{>}+\left|m\right|\right)!n_{<}!}{\left(n_{<}+\left|m\right|\right)!n_{>}!2^{|q-n|+|m|+1}}{\left[P_{n_{<}}^{\left(|q-n|,|m|\right)}\left(0\right)\right]}^{2}\\ & = & \frac{\left(n+|m|\right)!\left(q+|m|\right)!n!q!}{2^{n+q+|m|+1}}{\left[\sum_{k=0}^{n_{<}}\frac{{\left(-1\right)}^{k}}{k!(n-k)!(q-k)!(k+|m|)!}\right]}^{2}. \end{array}$$

**Figure 1:**
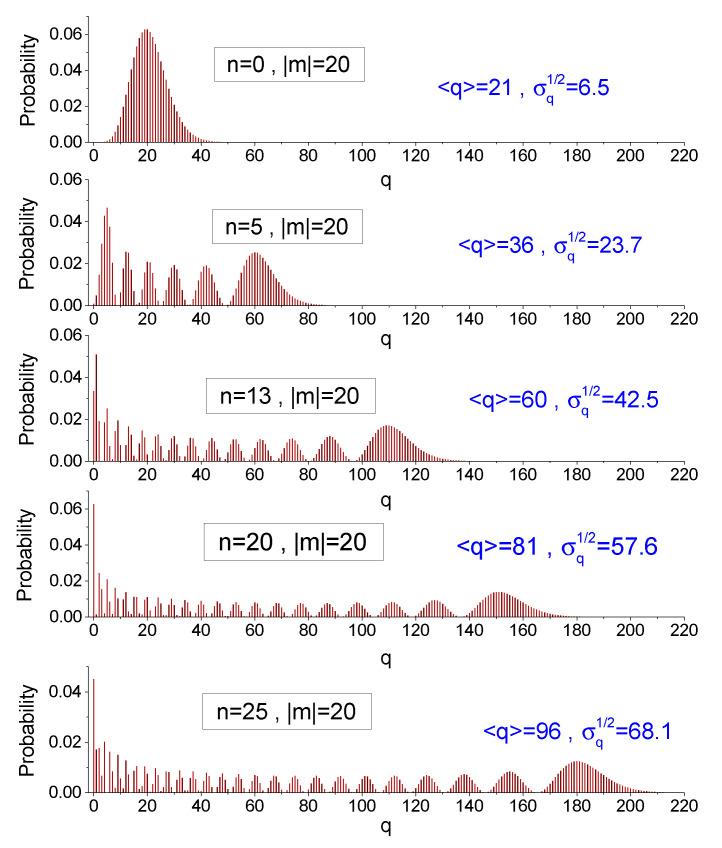
The probability distribution (44) of finding the initial Fock state |n,m〉 in the Fock state |q,m〉 after the frequency slowly passes through zero value, as a function of *q* for a fixed parameter *m*, in the case of $|u_{-}|=1$ and $|u_{+}|=\sqrt{2}$.

**Figure 2:**
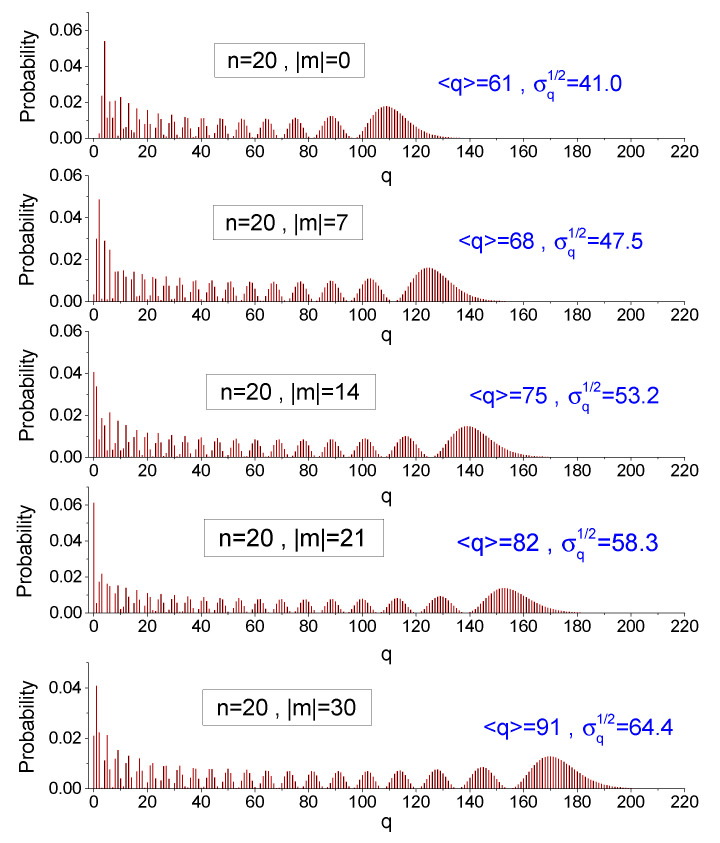
The probability distribution (44) of finding the initial Fock state |n,m〉 in the Fock state |q,m〉 after the frequency slowly passes through zero value, as a function of *q* for a fixed parameter *n*, in the case of $|u_{-}|=1$ and $|u_{+}|=\sqrt{2}$.

Several examples of the distribution (44) as a function of $q_{r}=q$ are shown in Figure 1 and Figure 2 for various values of parameters $n_{r}=n$ and |m|. We see that the distributions are very wide. Moreover, periodic structures are observed, and these structures become almost regular for large values of number *q*. The number of these structures is close to n+1 when parameters *n* and |m| are not too small. The symbols 〈q〉 and $\sigma_{q}^{1/2}$ stand for the average value of number *q* and mean square deviation of this number, calculated numerically.

**Figure 3:**
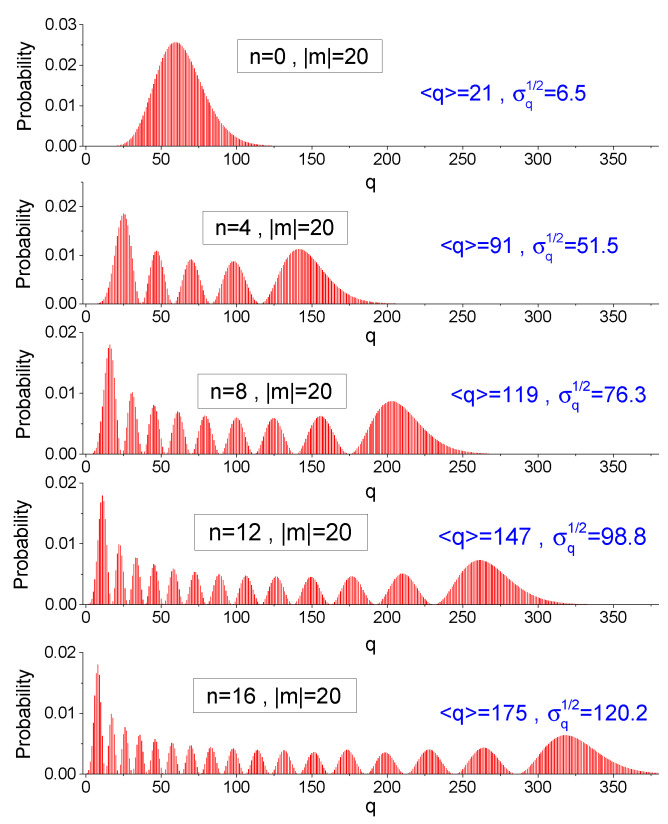
The probability distribution (44) of finding the initial Fock state |n,m〉 in the Fock state |q,m〉 after the frequency slowly passes through zero value, as a function of *q* for a fixed parameter *m*, in the case of $|u_{-}|=\sqrt{3}$ and $|u_{+}|=2$.

**Figure 4:**
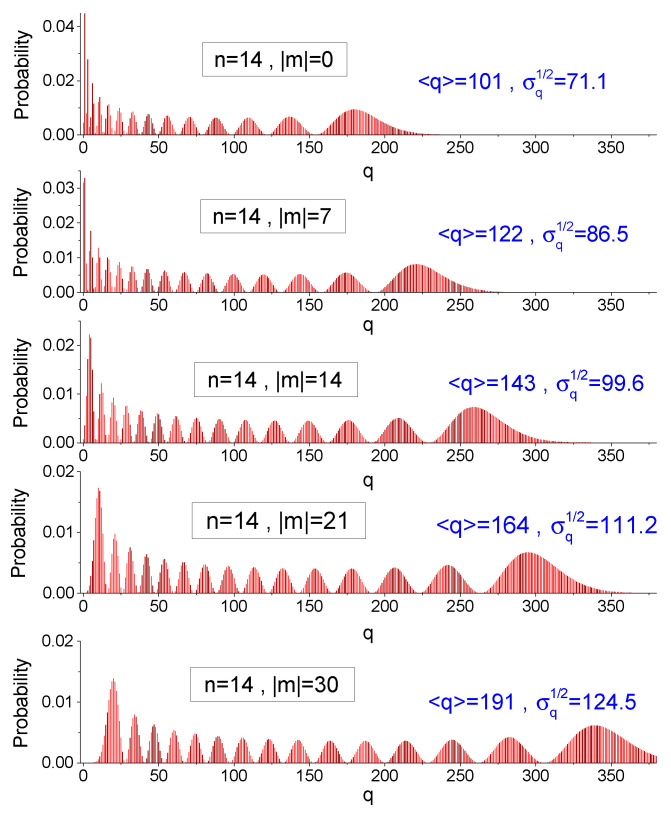
The probability distribution (44) of finding the initial Fock state |n,m〉 in the Fock state |q,m〉 after the frequency slowly passes through zero value, as a function of *q* for a fixed parameter *n*, in the case of $|u_{-}|=\sqrt{3}$ and $|u_{+}|=2$.

In Figure 3 and Figure 4, we show similar distributions for $|u_{-}|=\sqrt{3}$ and $|u_{+}|=2$. These values correspond to the “parabolic” form of function ω(t) near the point $t_{*}$, when the Larmor frequency attains zero; however, it does not change its sign, returning slowly to positive values. In this case,

(45) $$\begin{array}{ll} |C_{nq}^{\left(m\right)}{|}_{|u_{+}|=2}^{2} & =\frac{\left(n_{>}+\left|m\right|\right)!n_{<}!{\left(3/4\right)}^{\left|q-n\right|}}{\left(n_{<}+\left|m\right|\right)!n_{>}!4^{|m|+1}}{\left[P_{n_{<}}^{\left(|q-n|,|m|\right)}\left(-1/2\right)\right]}^{2}\\ & =\frac{\left(n+|m|\right)!\left(q+|m|\right)!n!q!3^{q+n}}{4^{|m|+1+q+n}}{\left[\sum_{k=0}^{n_{<}}\frac{{\left(-3\right)}^{-k}}{k!(n-k)!(q-k)!(k+|m|)!}\right]}^{2}. \end{array}$$

The plots of distribution (45) are similar to those for distribution (44), with the same number $n_{r}+1$ of periodic structures, but with an increased mean value 〈q〉. A simple analytic formula for this mean value is derived in Section 6.

**Figure 5:**
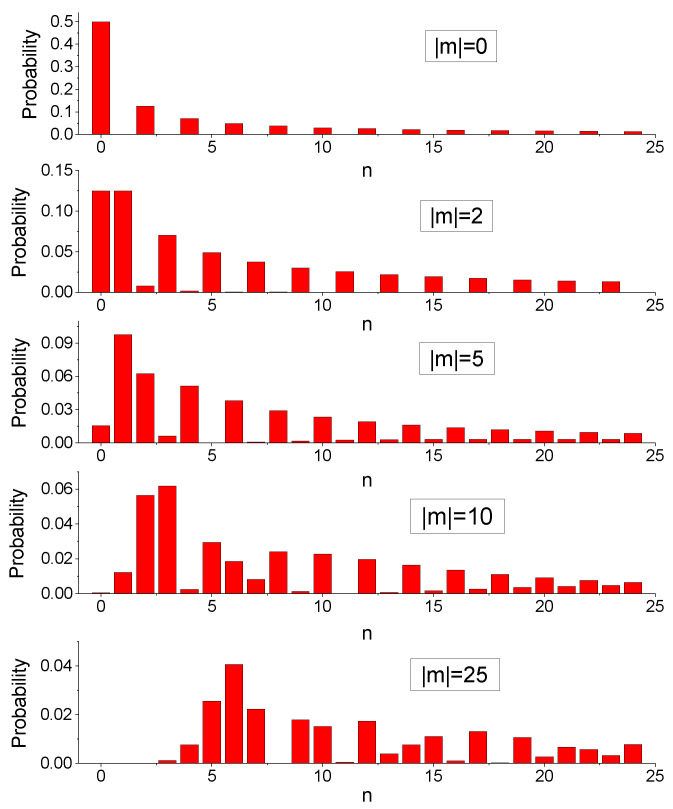
The probability of finding the initial Fock state |n,m〉 in the same Fock state after the frequency slowly passes through zero value, as a function of *n* for different fixed values of the angular moment quantum number |m|, for $|u_{-}|=1$ and $|u_{+}|=\sqrt{2}$.

**Figure 6:**
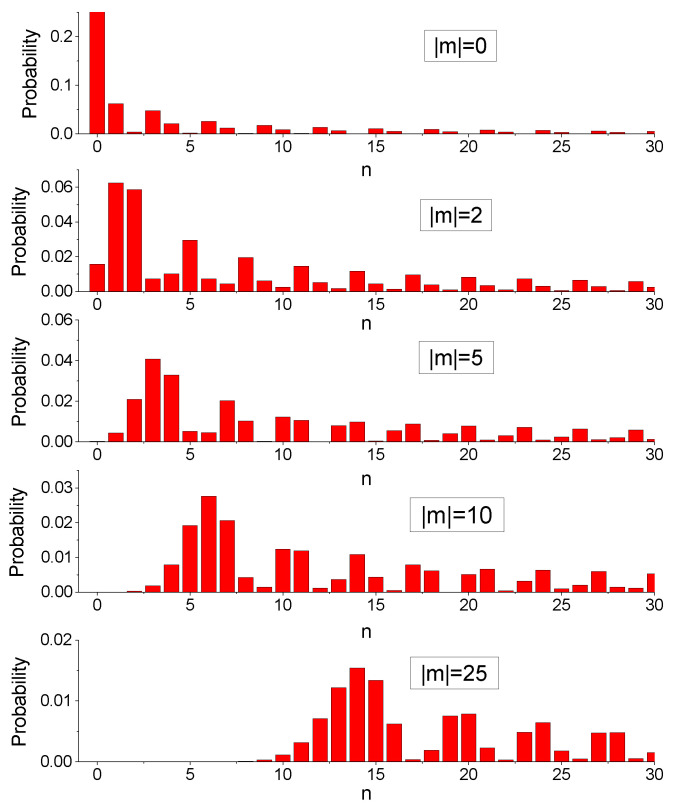
The probability of finding the initial Fock state |n,m〉 in the same Fock state after the frequency slowly passes through zero value, as a function of *n* for different fixed values of the angular moment quantum number |m|, for $|u_{-}|=\sqrt{3}$ and $|u_{+}|=2$.

The probability $|C_{nn}^{\left(m\right)}{|}^{2}$ of remaining in the initial Fock state turns out to be very low for many initial quantum numbers *n*, except for the case of n=m=0: see Figure 5 and Figure 6.

## 6. Mean Energy

To find the mean energy after the inversion of the magnetic field for an arbitrary initial energy eigenstate, one could try to calculate the sum

(46) $$\sum_{q_{r}=0}^{\infty }|C_{n_{r}q_{r}}^{\left(m\right)}{|}^{2}\left(1+\left|m\right|-\frac{\omega (t)}{\left|\omega (t)\right|}m+2q_{r}\right)=1+\left|m\right|-\frac{\omega (t)}{\left|\omega (t)\right|}m+2\sum_{q_{r}=0}^{\infty }{\left|C_{n_{r}q_{r}}^{\left(m\right)}\right|}^{2}q_{r}.$$

However, this method is not easy, because the sum contains squares of the Jacobi polynomials with variable lower and upper indexes. A direct calculation of the kinetic energy mean value $\langle {\hat{p}}_{x}^{2}+{\hat{p}}_{y}^{2}\rangle /2$ requires a knowledge of complicated integrals containing derivatives of function (6). It is probable that the simplest way is to notice that Hamiltonian (2) is quadratic with respect to the coordinates and momenta operators. Consequently, the Heisenberg or Ehrenfest equations for these operators are linear. This means that their time-dependent mean values are linear combinations of the initial values with certain time-dependent coefficients. It is convenient to introduce vector $Q=(x,y,p_{x},p_{y})$ (whose components are either mean values of quantum operators or classical variables). Then,

(47) $$Q\left(t\right)=\Lambda \left(t;t_{i}\right)Q\left(t_{i}\right),$$

where $\Lambda (t;t_{i})$ is a 4×4 matrix. Combining mean values $Q_{jk}\equiv \langle {\hat{Q}}_{j}{\hat{Q}}_{k}+{\hat{Q}}_{k}{\hat{Q}}_{j}\rangle /2$ into the 4×4 symmetric matrix Q, one can verify the relation (see [35])

(48) $$Q\left(t\right)=\Lambda \left(t;t_{i}\right)Q\left(t_{i}\right)\tilde{\Lambda }\left(t;t_{i}\right),$$

where $\tilde{\Lambda }$ means the transposed matrix. In our case, matrix $\Lambda (t;t_{i})$ can be expressed in terms of the solution ε(t) and its derivative $\dot{\varepsilon }\left(t\right)$ as follows [34],

(49) Λ(t;ti)=ωi1/2Re(ε)RIm(ε)R/ωiRe(ε˙)RIm(ε˙)R/ωi,R=cosϕ˜sinϕ˜−sinϕ˜cosϕ˜,

with the phase $\tilde{\phi }$ defined in Equation (11). Comparing Equations (2), (5) and (15), we obtain the initial mean value

$${\langle {\hat{p}}_{x}^{2}+{\hat{p}}_{y}^{2}\rangle }_{n_{r}m}=\omega_{i}\left(2n_{r}+\left|m\right|+1\right).$$

Moreover, since the initial wave function (3) possesses rotational symmetry, the initial covariance matrix $Q(t_{i})$ can be written in the following block form:

(50) $$Q\left(t_{i}\right)=\frac{\hbar }{2}∥\begin{array}{cc} I\gamma /\omega_{i} & m\Sigma \\ -m\Sigma & I\gamma \omega_{i} \end{array}∥,\;\;I=∥\begin{array}{cc} 1 & 0\\ 0 & 1 \end{array}∥,\;\;\Sigma =∥\begin{array}{cc} 0 & 1\\ -1 & 0 \end{array}∥,$$

where $\gamma =2n_{r}+\left|m\right|+1$. In this case, we obtain

(51) $$Q\left(t\right)=\frac{\hbar }{2}∥\begin{array}{cc} {I\gamma |\varepsilon \left(t\right)|}^{2} & m\Sigma +I\gamma Re\left(\dot{\varepsilon }\varepsilon^{*}\right)\\ -m\Sigma +I\gamma Re\left(\dot{\varepsilon }\varepsilon^{*}\right) & I\gamma |\dot{\varepsilon }{\left(t\right)|}^{2} \end{array}∥.$$

Consequently,

(52) $$\langle \hat{H}\left(t\right)\rangle =\frac{\hbar \gamma }{2}\left[|\dot{\varepsilon }{\left(t\right)|}^{2}+{\left|\varepsilon \left(t\right)\right|}^{2}\omega^{2}\left(t\right)\right]-m\hbar \omega \left(t\right).$$

In the adiabatic regime, using Equation (21), we obtain the formula

(53) $$\langle \hat{H}\left(t\right)\rangle =\hbar \left|\omega \left(t\right)\right|\left(2n_{r}+\left|m\right|+1\right)\left(|u_{+}{|}^{2}+{\left|u_{-}\right|}^{2}\right)-\hbar \omega \left(t\right)m,$$

which goes to (43) if $n_{r}=0$. Comparing Equations (24) and (25) with Equation (53), we see that Equation (19) can be generalized as follows,

(54) $$\bar{\langle M\rangle }=-\langle \hat{H}\left(t\right)\rangle /\left[\hbar \omega \left(t\right)\right],$$

where the overline means an additional time averaging over fast oscillations of the mean magnetic moment, as soon as $\bar{w(t)}=|u_{+}{|}^{2}+{\left|u_{-}\right|}^{2}$.

Comparing Equations (46) and (53), it is easy to see the equality

$$\left(1+2|u_{-}{|}^{2}\right)\left(2n_{r}+\left|m\right|+1\right)=2\langle q\rangle +\left|m\right|+1,$$

which results in the formula

(55) $$\langle q\rangle =n_{r}\left(1+2|u_{-}{|}^{2}\right)+|u_{-}{|}^{2}\left(|m|+1\right).$$

In particular, we have $\langle q\rangle =3n_{r}+\left|m\right|+1$ for $|u_{-}{|}^{2}=1$ and $\langle q\rangle =7n_{r}+3\left(|m|+1\right)$ for $|u_{-}{|}^{2}=3$. These relations coincide with the results of numeric calculations given in Figure 1, Figure 2, Figure 3 and Figure 4.

It is interesting to calculate the variance $\sigma_{H}=\langle {\hat{H}}^{2}\rangle -{\langle \hat{H}\rangle }^{2}$, characterizing the energy fluctuations. This variance equals zero in the initial Fock state. However, it becomes nonzero when the frequency passes through zero. Relatively simple calculations can be performed if $n_{r}=0$. Then, the sum

$$\sum_{q_{r}=0}^{\infty }{\left|C_{0q_{r}}^{\left(m\right)}\right|}^{2}{\left(1+\left|m\right|-\frac{\omega (t)}{\left|\omega (t)\right|}m+2q_{r}\right)}^{2}$$

can be found with the aid of Formula (42) and its consequences. The results are as follows,

$$\begin{array}{ccc} \langle {\hat{H}}^{2}\rangle & = & {\left[\hbar \omega \left(t\right)\right]}^{2}\left[{\left(1-\frac{\omega (t)}{\left|\omega (t)\right|}m+\left|m\right|\right)}^{2}+4\left(1+|m|\right)\left(2-\frac{\omega (t)}{\left|\omega (t)\right|}m+\left|m\right|\right){\left|u_{-}\right|}^{2}\right.\\ & & \left.+4(1+|m|)(2+|m|)|u_{-}{|}^{4}\right], \end{array}$$

(56) $$\sigma_{H}=4{\left[\hbar \omega \left(t\right)\right]}^{2}\left(1+|m|)\right|u_{+}u_{-}{|}^{2}.$$

Note that the variance (56) does not depend on the sign of quantum number *m*, while the mean value (53) is sensitive to this sign. If $n_{r}>0$, the calculations become rather cumbersome, so we do not perform them here. For explicit expressions in some special cases, one can consult papers [32,36].

## 7. Adiabatic Evolution of the “Invariant States” of the Magnetic Moment Operator

The stationary Fock states (3) are determined by integral parameters $n_{r}=0,1,2,\ldots$ and m=0,±1,±2,… In these normalized states, the energy and canonical angular momentum have definite values. In addition, the *mean value* of the magnetic moment operator (13) does not depend on time in these states. Recently, a wider family of *isotropic* states, possessing time-independent mean values of the magnetic moment operator for the time-independent Hamiltonian (2) and named as “magnetic moment invariant states”, was found in paper [37]. They are determined by two *continuous* positive parameters, $G_{+}\geq 1$ and $G_{-}\geq 1$, with the following nonzero mean values (provided ω>0):

(57) $$\langle \pi_{x}^{2}\rangle =\langle \pi_{y}^{2}\rangle =\langle \hat{H}\rangle =\hbar \omega G_{+},$$

(58) $$\langle \hat{x}{\hat{p}}_{y}\rangle =-\langle \hat{y}{\hat{p}}_{x}\rangle =\frac{\hbar }{4}\left(G_{-}-G_{+}\right),\;\langle y\pi_{x}\rangle =-\langle x\pi_{y}\rangle =\frac{\hbar }{2}G_{+},$$

(59) $$\langle p_{x}^{2}\rangle =\langle p_{y}^{2}\rangle =\omega^{2}\langle x^{2}\rangle =\omega^{2}\langle y^{2}\rangle =\frac{1}{4}\hbar \omega \left(G_{+}+G_{-}\right).$$

The solution (3) results in Formulas (57)–(59) with the *integral* positive coefficients

(60) $$G_{\pm }=1+2n_{r}+\left|m\right|\mp m.$$

It is known that there exist many quite different quantum states with the same mean values of canonical operators and their powers (products). In particular, one can construct a two-parameter family of *Gaussian* states possessing the second-order moments (57)–(59) with arbitrary coefficients $G_{\pm }\geq 1$. Their Wigner functions have the following form [37] [here **r** = (x,y) and $p=(p_{x},p_{y})$]:

(61) $$W_{G}\left(r,p\right)=\frac{4}{G_{+}G_{-}}\exp\left\{-\;\frac{\left(G_{+}+G_{-}\right)\left[\omega^{2}r^{2}+p^{2}\right]+2\left(G_{+}-G_{-}\right)\omega \left(xp_{y}-yp_{x}\right)}{2\hbar \omega G_{+}G_{-}}\right\}.$$

The quantum purity of the state (61) equals

(62) $$P=\int W^{2}\left(r,p\right)\frac{drdp}{{\left(2\pi \hbar \right)}^{2}}={\left(G_{+}G_{-}\right)}^{-1}.$$

Consequently, all Gaussian “magnetic moment invariant states” are *mixed* if $G_{\pm }>1$, in contradistinction to the *pure* quantum states (3) with integral values of parameters $G_{\pm }$, given by Equation (60). The meaning of parameters $G_{+}$ and $G_{-}$ becomes more clear, if one goes from the canonical operators in the phase space to the relative $\left(x_{r},y_{r}\right)$ and guiding center $\left(x_{c},y_{c}\right)$ coordinates,

(63) $$x_{r}=-\pi_{y}/\left(2m\omega \right),\;\;\;y_{r}=\pi_{x}/\left(2m\omega \right),\;\;\;\;\;x_{c}=x+\pi_{y}/\left(2m\omega \right),\;\;\;y_{c}=y-\pi_{x}/\left(2m\omega \right).$$

The importance of these integrals of motion was emphasized by many authors in recent decades [24,38,39,40,41,42,43,44,45,46,47,48,49,50,51,52,53,54,55]. Equivalent integrals of motion, obtained by the multiplication of $x_{c}$ and $y_{c}$ by mω, were considered under the name “pseudomomentum” in papers [43,56,57].

The corresponding second-order moments are as follows,

(64) $$\langle x_{r}^{2}\rangle =\langle y_{r}^{2}\rangle =\frac{\hbar }{4\omega }G_{+},\;\langle x_{c}^{2}\rangle =\langle y_{c}^{2}\rangle =\frac{\hbar }{4\omega }G_{-},$$

with zero mean values of all cross-products. Now, the inequalities $G_{\pm }\geq 1$ follow from the commutators $\left[{\hat{x}}_{r},{\hat{y}}_{r}\right]=\left[{\hat{y}}_{c},{\hat{x}}_{c}\right]=i\hbar /\left(2\omega \right)$ and the Heisenberg–Weyl (actually, Robertson’s [58]) uncertainty relation

(65) $$\langle A^{2}\rangle \langle B^{2}\rangle \geq {\left|\langle \left[\hat{A},\hat{B}\right]\rangle \right|}^{2}/4.$$

The evolution of the second-order moments can be calculated in the same way as that shown in Section 6. It is sufficient to replace γ with $\left(G_{+}+G_{-}\right)/2$ and *m* with $\left(G_{-}-G_{+}\right)/2$ in Equations (50) and (51). Hence, the mean values of energy and magnetic moment for $t>t_{i}$ can be written as follows,

(66) $$E\left(t\right)\equiv \langle \hat{H}\left(t\right)\rangle =\frac{\hbar }{4}\left(G_{+}+G_{-}\right)\left[|\dot{\varepsilon }{\left(t\right)|}^{2}+\omega^{2}\left(t\right){\left|\varepsilon \left(t\right)\right|}^{2}\right]+\frac{\hbar }{2}\omega \left(t\right)\left(G_{+}-G_{-}\right),\;$$

(67) $$M\left(t\right)=\frac{\mu_{B}}{2}\left[G_{-}-G_{+}-\omega \left(t\right){\left|\varepsilon \left(t\right)\right|}^{2}\left(G_{+}+G_{-}\right)\right].$$

If the frequency slowly goes to a constant asymptotic value $\omega_{f}$ (which can be positive or negative), then the quantum mechanical mean value of the energy tends to a time-independent value

(68) $$E_{f}=\frac{\hbar }{2}\left[|\omega_{f}|\left(G_{+}+G_{-}\right)\left(1+2|u_{-}{|}^{2}\right)+\omega_{f}\left(G_{+}-G_{-}\right)\right].$$

The quantum-mechanical mean value of the kinetic angular momentum performs harmonic oscillations with frequency $2|\omega_{f}|$ between the values $M_{min}$ and $M_{max}$. The amplitude of these oscillations equals

(69) $$\Delta M=\frac{1}{2}\left(M_{max}-M_{min}\right)=\mu_{B}\left|u_{+}u_{-}\right|\left(G_{+}+G_{-}\right).$$

The time-averaged value of these oscillations equals

(70) $$\bar{\langle M\rangle }=\frac{\mu_{B}}{2}\left[G_{-}-G_{+}-\frac{\omega_{f}}{|\omega_{f}|}\left(G_{+}+G_{-}\right)\left(1+2|u_{-}{|}^{2}\right)\right].$$

## 8. Conclusions

The main results of this paper include the discovery of the existence of generalized adiabatic invariants and a generalized Born–Fock adiabatic theorem for a charged particle in a homogeneous magnetic field, when this field slowly passes through zero value. According to the original Born–Fock theorem, the initial discrete energy eigenstates maintain their forms during the adiabatic evolution of the Hamiltonian, so that parameters of the wave functions correspond to instantaneous values of coefficients of the Hamiltonian. This statement becomes invalid when the time-dependent Larmor frequency approaches zero value. We considered a scenario where the frequency slowly departs from zero, in such a way that the adiabatic condition (20) is reestablished. The evolved quantum state then becomes a *superposition* of instantaneous energy eigenstates with different radial quantum numbers (but the same angular quantum numbers, due to the conservation of the canonical angular momentum). The generalized Born–Fock theorem states that the weight of each member of this superposition does not depend on time, as soon as the condition (20) is fulfilled. It is remarkable that all these weights depend on the single parameter $|u_{-}|$ of the most general adiabatic solution (21) to the classical Equation (7) of the harmonic oscillator with a time-dependent frequency. Using examples of exact solutions, we believe (although we have no rigorous proof) that the value of $|u_{-}|$ in the case of a single transition of frequency through zero is determined by the exponent *k* in the frequency behavior near zero: $\omega \left(t\right)∼{\left(t-t_{*}\right)}^{k}$. In particular, $|u_{-}|=1$ if k=1. Regarding cases where there are multiple frequency passages through zero, the final coefficient $|u_{-}|$ is highly sensitive to the additional parameter—the phase Φ, which is the integral of the absolute value |ω(t)| between the first and last zero frequency instants. Consequently, the adiabatic behavior after many crossings through zero frequency can be quasi-chaotic. Under certain specific conditions, the mean energy can even return to the initial value after multiple frequency passage through zero, while it can be significantly amplified under other specific conditions. However, in all the cases, the adiabatic ratio $\langle \hat{H}\left(t\right)\rangle /\left[\hbar |\omega \left(t\right)\right]$ can only increase after many frequency passages through zero value. This ratio coincides (by absolute value) with the double mean value of the magnetic moment in the adiabatic regime. The word “double” means the quantum-mechanical averaging accompanied with the time averaging over rapid oscillations in time.

Note that the concepts of adiabaticity and multiple passages through zero frequency are completely compatible. For example, the Larmor frequency of an electron in the magnetic field of the order of 1 T is of the order of $10^{11}$ s${}^{-1}$. In such a case, periodic variations of the magnetic field with the standard frequency 50 Hz are totally adiabatic. It would be interesting to observe what can happen with the statistical properties of the energy and magnetic moment in this realistic situation after a great number (of an order of $10^{2}$ or higher) of frequency transitions through zero. However, this can be the subject of another study. There are several other challenging problems. In particular, in the case of very slow evolution, various *dissipation* effects can change the evolution drastically. Another problem to address is gaining an understanding of the adiabatic evolution for different gauges of the vector potential. Some results presented in the paper by Ref. [36] indicate that the evolution can be quite different for the Landau gauge, because of another geometry of the induced electric field. However, the general linear gauge of the time-dependent magnetic field remains an unsolved problem. Furthermore, we pose the following question: What happens to arbitrary superpositions of the Fock states in the case of adiabatic evolution, especially when the magnetic field changes its sign? We see that the number of unsolved interesting problems is not small.

## Data Availability

Not applicable.

## Appendix A. Integrals with Squares of the Laguerre Polynomials

Since ${\left|\exp\left[ir^{2}\dot{\varepsilon }/\left(2\hbar \varepsilon \right)\right]\right|}^{2}=\exp\left(-Kr^{2}\right)$, due to identity (9), the calculation of the mean values $\langle r^{2j}\rangle$ in the state (6) can be reduced (using the substitution $x=Kr^{2}$) to the calculation of the integrals

(A1) $$I_{j}=\int_{0}^{\infty }x^{a+j}e^{-x}{\left[L_{n}^{\left(a\right)}\left(x\right)\right]}^{2}dx.$$

These integrals are absent in the available reference books (such as [4], for example). However, it is easy to see that $I_{1}=-\lim_{b\to 1}\partial J\left(b\right)/\partial b$, where J(b) is the special case of a more general integral 7.414.4 from [4]:

(A2) $$\begin{array}{ccc} J(b) & = & \int_{0}^{\infty }x^{a}e^{-bx}{\left[L_{n}^{\left(a\right)}\left(x\right)\right]}^{2}dx\\ & = & \frac{\left(2n+a\right)!{\left(b-1\right)}^{2n}}{{\left(n!\right)}^{2}b^{2n+a+1}}F\left[-n,-n;-2n-a;\frac{b(b-2)}{{\left(b-1\right)}^{2}}\right]. \end{array}$$

Here, F(u,v:w;z) is the Gauss hypergeometric function. Using the definition of this function, we can write

(A3) $$\begin{array}{ccc} J(b) & = & b^{-2n-a-1}\left\{\frac{(n+a)!}{n!}{\left[b\left(2-b\right)\right]}^{n}+{\left(b-1\right)}^{2}{\left[b\left(2-b\right)\right]}^{n-1}\frac{(n+a+1)!}{(n-1)!}\right.\\ & & \left.+{\left(b-1\right)}^{4}f\left(b\right)\right\}, \end{array}$$

where f(b) is some function, which is regular at the point b=1. Calculating the derivative of function (A3) with respect to *b* and putting b=1 in the final expression, we arrive at the formula

(A4) $$I_{1}=\frac{(n+a)!}{n!}\left(2n+a+1\right).$$

Its consequence is Formula (15). Similarly,

(A5) $$I_{2}=\lim_{b\to 1}\partial^{2}J\left(b\right)/\partial b^{2}=\frac{(n+a)!}{n!}\left[6n(n+a+1)+(a+1)(a+2)\right].$$

## Appendix B. Calculation of Coefficients in the Expansion (39)

The scalar product $\langle \psi_{q_{r}m}|\Psi_{n_{r}m}\left(t\right)\rangle$ can be calculated with the help of Equation 7.414.4 from [4],

(A6) $$\begin{array}{ccc} I & = & \int_{0}^{\infty }x^{a}e^{-bx}L_{n}^{\left(a\right)}\left(\lambda x\right)L_{q}^{\left(a\right)}\left(\mu x\right)dx\\ & = & \frac{\left(n+q+a\right)!{\left(b-\lambda \right)}^{n}{\left(b-\mu \right)}^{q}}{n!q!b^{n+q+a+1}}F\left[-n,-q;-n-q-a;\frac{b(b-\lambda -\mu )}{\left(b-\lambda )(b-\mu \right)}\right]. \end{array}$$

The Gauss hypergeometric function in the right-hand side is a polynomial of the order $n_{<}=min\left(n,q\right)$. One of possible forms is the expression in terms of the Jacobi polynomial (see Equation 4.22.1 from [59]):

(A7) $$P_{n}^{\left(\alpha ,\beta \right)}\left(x\right)=\frac{(2n+\alpha +\beta )!}{n!(n+\alpha +\beta )!}{\left(\frac{x-1}{2}\right)}^{n}F\left(-n,-n-\alpha ;-2n-\alpha -\beta ;\frac{2}{1-x}\right).$$

Then, Formula (A6) can be written as follows (assuming q≥n),

(A8) $$I=\frac{\left(q+|m|\right)!{\left(b-\mu \right)}^{q-n}{\left(\lambda +\mu -b\right)}^{n}}{q!b^{q+|m|+1}}P_{n}^{\left(q-n,|m|\right)}\left(\frac{b(\lambda +\mu -b)-2\lambda \mu }{b(b-\lambda -\mu )}\right).$$

In our case, we have $x=r^{2}$ and λ=|ω|/ℏ. Other parameters are as follows,

$$b=\frac{1}{2\hbar }\left(\left|\omega \right|-\frac{i\dot{\varepsilon }}{\varepsilon }\right)=\frac{\left|\omega \right|u_{+}e^{i\phi }}{\hbar z},\;\mu =\frac{\left|\omega \right|}{{\hbar |z|}^{2}},\;z=u_{+}e^{i\phi }+u_{-}e^{-i\phi },$$

$$b-\lambda =-\;\frac{\left|\omega \right|u_{-}e^{-i\phi }}{\hbar z},\;b-\mu =\frac{\left|\omega \right|u_{-}^{*}e^{i\phi }}{\hbar z^{*}},\;\lambda +\mu -b=\frac{\left|\omega \right|u_{+}^{*}e^{-i\phi }}{\hbar z^{*}}.$$

The phase ϕ was defined in Equation (21). Consequently, the argument of the Jacobi polynomial in Equation (A8) equals

$$\frac{b(\lambda +\mu -b)-2\lambda \mu }{b(b-\lambda -\mu )}=\frac{1-|u_{-}{|}^{2}}{1+|u_{-}{|}^{2}}.$$

Combining all terms in the Formulas (3), (6) and (A8), we obtain the following expression for the complex coefficient $\langle \psi_{q_{r}m}|\Psi_{n_{r}m}\left(t\right)\rangle$ with $q_{r}\geq n_{r}$:

(A9) $$\begin{array}{ccc} \langle \psi_{q_{r}m}|\Psi_{n_{r}m}\left(t\right)\rangle & = & {\left[\frac{(q_{r}+|m|)!n_{r}!}{(n_{r}+|m|)!q_{r}!}{\left(\frac{z}{z^{*}}\right)}^{2q_{r}+\left|m\right|+1}\right]}^{1/2}e^{i\chi \left(t\right)-i\phi \left(t\right)(2n_{r}+|m|+1)}\\ & & \times \frac{{\left(u_{-}^{*}\right)}^{q_{r}-n_{r}}{\left(u_{+}^{*}\right)}^{n_{r}}}{u_{+}^{q_{r}+\left|m\right|+1}}P_{n_{r}}^{\left(q_{r}-n_{r},|m|\right)}\left(\frac{1-|u_{-}{|}^{2}}{1+|u_{-}{|}^{2}}\right). \end{array}$$

Similar formulas (in a slightly different context) were found in paper [5]. All phase factors disappear when one calculates the module squared $|\langle \psi_{q_{r}m}|\Psi_{n_{r}m}\left(t\right)\rangle {|}^{2}$. Moreover, this quantity becomes symmetric with respect to quantum numbers $q_{r}$ and $n_{r}$. The result is given by Equation (40) of the main text.
